# Is It Possible to Screen for Primary Aldosteronism Effectively in Primary Care?

**DOI:** 10.1111/cen.15247

**Published:** 2025-04-07

**Authors:** Harsha Anuruddhika Dissanayake, Bronwen Warner, Anne‐Marie Hannon, Riccardo Pofi, Radu Mihai, Tim James, Brian Shine, David William Ray, Jeremy W. Tomlinson, Aparna Pal

**Affiliations:** ^1^ Oxford Centre for Diabetes, Endocrinology & Metabolism Churchill Hospital Oxford UK; ^2^ Department of Clinical Medicine, Faculty of Medicine University of Colombo Colombo Sri Lanka; ^3^ Department of Surgery and Cancer Imperial College London UK; ^4^ Department of Endocrinology University Hospital Limerick Limerick Ireland; ^5^ Oxford Centre for Diabetes, Endocrinology & Metabolism, NIHR Oxford Biomedical Research Centre University of Oxford Oxford UK; ^6^ Department of Endocrine Surgery, Churchill Cancer Centre Oxford University Hospitals NHS Foundation Trust Oxford UK; ^7^ Department of Clinical Biochemistry Oxford University Hospitals NHS Foundation Trust Oxford UK; ^8^ NIHR Oxford Health Biomedical Research Centre, and NIHR Oxford Biomedical Research Centre John Radcliffe Hospital Oxford UK; ^9^ Oxford Centre for Diabetes, Endocrinology and Metabolism, and Oxford Kavli Centre for Nanoscience Discovery University of Oxford Oxford UK

**Keywords:** case detection, endocrine hypertension, general practice, hyperaldosteronism, resistant hypertension

## Abstract

**Objective:**

Primary aldosteronism (PA) is the commonest secondary cause of hypertension but case‐detection remains a challenge. Screening is usually performed in secondary care using an aldosterone:renin ratio (ARR) measurement. Here, we describe the outcomes of screening in primary care, in Oxfordshire, UK.

**Design:**

Retrospective observational study.

**Patients:**

Adults screened for PA in primary care services in Oxford between 2008 and 2022.

**Measurements:**

ARR test results in primary care and outcomes of secondary care evaluation (ARR, saline infusion test, final diagnosis). Primary care and secondary care ARR tests were compared for correlation, concordance and performance in predicting PA.

**Results:**

Among 2915 adults screened in primary care, 455 were referred to secondary care and 107 (3.7% of total population screened) were diagnosed with PA. Primary care ARR showed strong correlation with secondary care ARR (*r* = 0.841, *p* < 0.001). Area under the ROC curve to predict PA was 0.81 (95% CI 0.77–0.86) for primary care ARR testing. Primary care ARR cut‐off of ≥ 30 pmol/mU showed comparable sensitivity (91.7% vs 92.1%, *p* = 0.467) to and modest concordance (Kappa 0.583, *p* < 0.001) with secondary care ARR. Use of beta‐blockers were associated with higher risk of false positive test result (OR 3.5, 95% CI 1.1–12.0, *p* = 0.042).

**Conclusions:**

Screening for PA in primary care with ARR is feasible with modest concordance and comparable sensitivity to secondary care testing. Simple referral criteria and raising awareness among primary care colleagues could ensure appropriate referral to secondary care.

## 1 Introduction

Primary aldosteronism (PA) is the commonest secondary cause for hypertension accounting for 10–15% of patients with hypertension and at least 25% of patients with resistant hypertension [1, 2, 3]. Hyperaldosteronism increases the risk of cardiovascular and renal disease via blood pressure dependent and independent mechanisms [4, 5, 6]. However, it remains under‐recognised, largely due to insufficient screening [7, 8, 9, 10].

Aldosterone to renin ratio (ARR) is the most widely used screening test for PA [2]. In the UK, this is traditionally done via secondary care services. The potential role of primary care screening for PA is increasingly recognised [11, 12] and has been reported in several other countries with detection rates ranging from 0.7% to 14% [13, 14]. This variability is at least partly due to heterogeneity in screening criteria, test methodology and use of different cut‐off thresholds.

Primary care PA screening has been available in Oxfordshire since 2008. We aimed to analyse the primary care PA screening practices, referral pathways, PA detection rate and correlation and concordance between primary care and secondary care screening.

## 2 Materials and Methods

A retrospective observational study was conducted by screening the database of the Biochemistry Department and Electronic Patient Records at Oxford University Hospitals (OUH). The study was registered and approved as an audit in the OUH Trust (Audit number: 7174).

Primary care screening was initiated with blood sampling in the respective primary care centres and samples sent to the reference laboratory in Oxford University Hospitals. Samples are transported to the reference laboratory within 4 h of collection and delayed samples are rejected and notified so that re‐sampling can be arranged. Those with abnormal ARR were referred as deemed necessary to secondary care and many of those patients had ARR repeated in secondary care at the same reference laboratory. To minimise interference with ARR test interpretation, antihypertensives were converted to alpha blockers or long‐acting calcium channel blockers before repeating ARR in secondary care and before confirmatory testing, in line with international guidelines [2]. When this was not possible or not adhered to, results were interpreted in context of antihypertensive medications used at the time of testing by cross checking with primary care medication records.

From 2008 to 2015, aldosterone concentration (pmol/L) was measured using immunoassay and renin (pmol/L/hour) was determined using an activity assay in a reference laboratory [15]. After 2015, aldosterone (pmol/L) and renin (mU/L) were measured using IDS i‐Sys immunoassay methods (Immunodiagnostic Systems Limited, Boldon, UK) [16] locally at the Oxford University Hospitals Biochemistry Department. Recommended ARR cut offs deemed positive were ≥ 1000 h^−1^ before 2015 and ≥ 30 pmol/mU after 2015. For combined analyses, we used the following conversions for aldosterone and renin levels: [aldosterone level in post‐2015 assay in pmol/L] = 1.061 x [Aldosterone level in pre‐2015 assay in pmol/L] – 58.24. Similarly, [direct renin concentration in mU/L in post‐2015 assay] = 23.80 x [plasma renin concentration in pmol/L/hour in pre‐2015 assay] – 1.65 (unpublished data from departmental method comparisons).

All individuals who underwent ARR testing via primary care between 2008 and 2022 were identified from the biochemistry laboratory sample database. Electronic patient records were screened to identify if referral to secondary care had occurred and if so, what the final outcome was. Where an individual had more than one ARR in primary care, the most recent test before secondary care assessment was included in the analysis. Where an individual had more than 1 secondary care ARR, the most recent test, before saline infusion test or before implementation of definitive treatment, was included in the analysis.

A saline infusion test for confirmation of PA was performed in secondary care. It was conducted in the morning, whilst the patient was seated in a semi‐reclined chair. In line with Endocrine Society guidelines for diagnosis and management of PA [2], diagnosis of PA was considered confirmed (confirmed PA) in the following scenarios:

– aldosterone level ≥ 280 pmol/L post‐saline infusion test, OR
– aldosterone raised above 550 pmol/L, undetectable renin and spontaneous hypokalaemia OR
– aldosterone level 140–279 pmol/L post‐saline infusion test with strong clinical suspicion of PA, completed further evaluation and appropriate management.

The diagnosis of PA was considered possible if an individual had clinical features of PA and positive ARR (with or without aldosterone 140–279 pmol/L post saline infusion test) but not investigated further as surgery was not considered appropriate and therefore was managed with medical treatment. The diagnosis of PA was excluded if aldosterone level post saline infusion test was < 140 pmol/L or if the saline infusion test was not indicated due to negative result on repeat ARR and low clinical suspicion of PA.

Statistical analysis was performed using IBM SPSS Statistics for Windows, version 20 (IBM Corp., Armonk, N.Y., USA) and R statistical software (version 4.3.1., R core team, 2023). Continuous variables are expressed as mean and standard deviation for normally distributed variables and median and interquartile range for non‐normally distributed variables. Categorical variables are expressed as number and percentage. Correlations between non‐normally distributed variables were determined using the Spearman rank test. Between group comparisons of categorical variable were made using Chi square test. Concordance between primary and secondary care ARR tests was determined using the Kappa test. Performance of primary and secondary care ARR for diagnosis of confirmed PA were compared using DTCompAir package in R [17]. We used McNemar's test for comparison of sensitivity and specificity, relative predictive values for comparison of positive and negative predictive values, and diagnostic likelihood ratio regression model for comparison for positive and negative diagnostic likelihood ratios. Predictors of PA and determinants of false positive and false negative primary care ARR test results were determined using logistic regression models. Variables in the models were identified through previous literature [2] and conducting univariable regression analyses in our data set. Age, sex, presence of hypertension, number of antihypertensives used, hypokalaemia and aldosterone:renin ratio were included in the model for predicting PA. Age, sex and antihypertensive medication class(es) used were included in the models for predicting false positive (as opposed to true positive) and false negative (as opposed to true negative) primary care ARR test result. Where linearity assumption for continuous variables was violated, natural log transformations were used in regression models. Area under ROC curves (AUC) were used to compare predictive ability of biochemical tests in diagnosing PA. AUCs of tests in the two settings were compared using MedCalc software Ltd (Ostend, Belgium) as previously described [18, 19]. Optimal cut offs of diagnostic tests were determined using the Youden index. *p* value < 0.05 was considered significant. Missing data were excluded case‐wise from analyses.

## 3 Results

### 3.1 Primary Care Screening Practices and Population Characteristics

**Figure 1:**
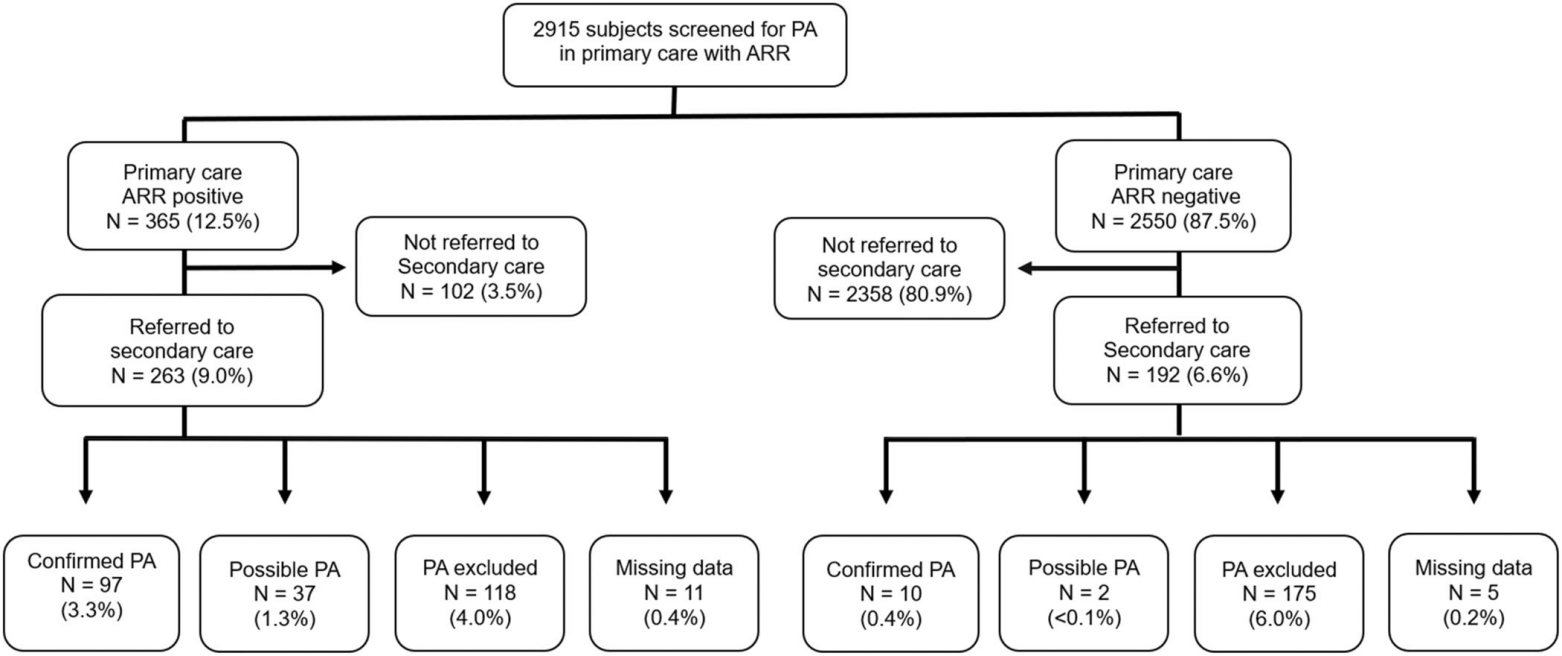
Outcomes of people screened for primary aldosteronism in primary care. All percentages are out of total population screened (*N *= 2915). Positive test was defined as ARR ≥ 1000 h^−1^ before 2015 and ≥ 30 pmol/mU after 2015. Data on final diagnosis was not available from 16 individuals due to on‐going investigations (*n *= 6) or lost to follow up/unavailability of data in electronic records (*n *= 10). ARR, aldosterone renin ratio; PA, primary aldosteronism; PC, primary care; SC, secondary care.

Between 2008 and 2022, 2915 individuals had the ARR test performed in primary care. Among them, 365 (12.5%) had a positive test result (Figure 1). Following primary care testing, 455 were referred to secondary care for further assessment (263/365 [72.1%] with a positive primary care ARR and 192/2550 [7.5%] with a negative primary care ARR). Among those referred, data on diagnostic evaluation and outcome were available for 439 individuals (Tables 1 and 2).

**Table 1:** Characteristics [median (IQR), mean ± SD, number (%)] of the people screened for primary aldosteronism in both primary and secondary care (*n *= 455).

Parameter	Value
Number of individuals	455
Age in years	50 (38–61)
Males	254 (55.8)
Antihypertensive medication use at the time of primary care ARR testing^a^	
None	114 (25.1)
1	125 (27.5)
2	65 (14.1)
3	12 (2.6)
4	7 (1.5)
Antihypertensives used at the time of primary care ARR testing a	
Mineralocorticoid receptor antagonist	11 (3.4)
ACE inhibitors/Angiotensin receptor blocker	85 (16.3)
Beta blocker	32 (9.9)
Dihydropyridine calcium channel blocker	95 (29.4)
Non‐dihydropyridine calcium channel blocker	2 (0.6)
Alpha blocker	68 (21.1)
Thiazide diuretic	28 (8.7)
Potassium level at primary care screening in mmol/L	3.5 ± 0.5
Indication for screening	
Hypertension with hypokalaemia	237 (52.1)
Hypertension/uncontrolled hypertension	39 (8.6)
Adrenal incidentaloma and hypertension	19 (4.2)
Adrenal incidentaloma	12 (2.6)
Young‐onset hypertension	76 (16.7)
Hypokalaemia	69 (15.2)
Unclear	3 (0.7)

*Note:* Medication use among those who had positive and negative primary care ARR tests are compared in Supporting Table S1.

^a^ Medication data available in 323 patients. Different patients were on different numbers and combinations of antihypertensives.

**Table 2:** Results [median (IQR), *n* (%)] of primary care screening, referral to secondary care and diagnostic outcomes for primary aldosteronism (*n* = 455).

Parameter	Value
Aldosterone level in pmol/L	459.0 (302.5–744.0)
Renin level in mU/L	13.6 (3.1–34.8)
Aldosterone to renin ratio pmol/mU	37.9 (5.7–71.7)
Secondary care service referred to	
Cardiology	81 (17.8)
Endocrine surgery	1 (0.2)
Endocrinology	352 (77.4)
Nephrology	20 (4.4)
External	1 (0.2)
Final confirmatory diagnosis	
PA excluded	293 (64.4)
Possible PA	39 (8.6)
Confirmed PA	107 (23.5)
Unknown^a^	16 (3.5)

Abbreiviation: PA, primary aldosteronism.

^a^ Final diagnosis unknown due to missing data/lost to follow‐up (*n *= 10) or on‐going evaluation (*n *= 6).

### 3.2 Outcome of Primary Care Screening

In the population screened in primary care (n = 2915), confirmed PA and possible PA were diagnosed in 107 (3.7%) and 39 (1.3%) individuals respectively. Among individuals referred to secondary care with a positive primary care ARR, confirmed PA and possible PA was diagnosed in 36.9% and 14.1% respectively. Among individuals referred with a negative primary care ARR, the respective prevalences were 5.2% and 1.1%. Outcomes of all individuals referred to secondary care are summarised in Figure 1.

**Figure 2:**
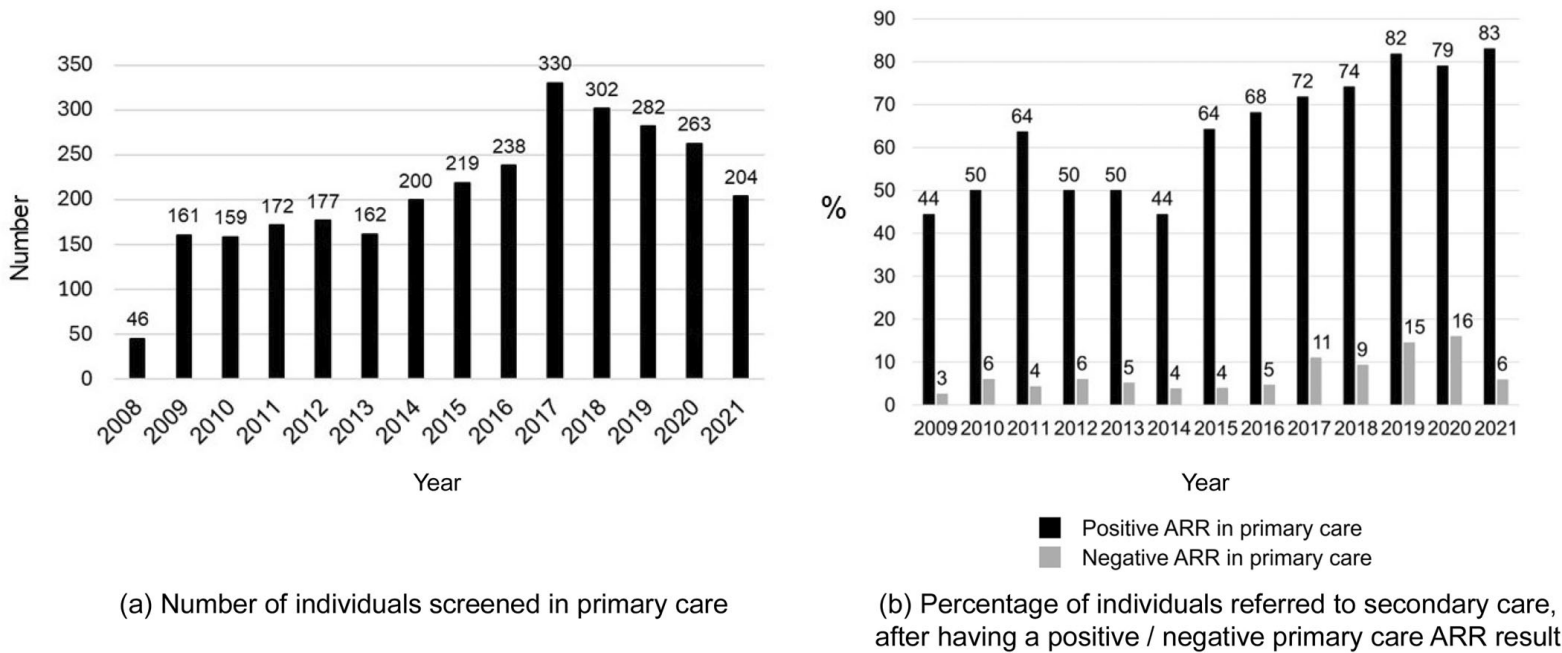
Primary care screening patterns over time. (a) Number of individuals screened for primary aldosteronism PA in primary care (PC) with aldosterone ratio (ARR) test (*N *= 2915). (b) Percentage of primary care screened individuals referred to secondary care (in total, 263 of 365 with a positive primary care ARR and 192 of 2550 with a negative primary care ARR were referred). Positive test was defined as ARR ≥ 1000 h^−1^ before 2015 and ≥ 30 pmol/mU after 2015.

The number of individuals screened in primary care increased from 2008 to 2017 and has decreased since then (Figure 2a). Overall, 72% of individuals with positive primary care screening were referred to secondary care. The percentage referred also increased over time (Figure 2b).

**Figure 3:**
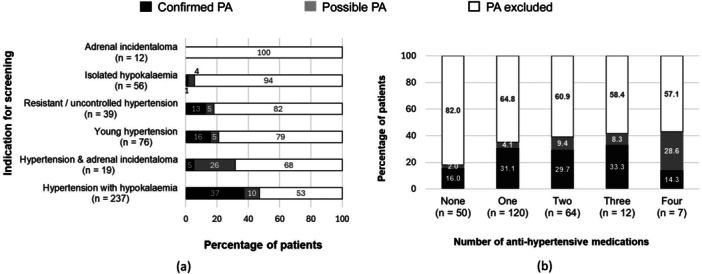
Percentage of patients diagnosed with confirmed or possible PA (a) across different indications [among 439 patients whose outcome of diagnostic evaluation was known], and, (b) by the number of antihypertensive medications used [among 253 individuals whose outcome of diagnostic evaluation and medication data were known]. Number of antihypertensive medications were determined by reviewing the prescription at the time of primary care testing. Among 50 individuals who were not on antihypertensives at the time of primary care ARR test, 13 were commenced on antihypertensive medication after the ARR test. PA, primary aldosteronism.

The prevalence of PA was low among individuals without hypertension (e.g., adrenal incidentaloma, hypokalaemia without hypertension) (Figure 3a). Among individuals with hypertension, the prevalence was higher among those who required at least one antihypertensive medication. However, we found that the number of antihypertensive medications was not of discriminatory value (Figure 3a, b).

Concordance between primary care and secondary care ARR test results were determined using data from individuals who had ARR test performed in both settings. Among 455 individuals referred to secondary care, ARR test results from both settings were available from 394 individuals. Among them, there was modest concordance between primary and secondary care test results (kappa = 0.583, *p* < 0.001, Supporting Figures S1 and S2).

Comparison of test performance (sensitivity and negative predictive value) was made using data from individuals who had a diagnosis of PA confirmed or PA excluded, and ARR tests in both primary and secondary care settings. Among the 455 individuals referred to secondary care, 400 individuals had a confirmed outcome (confirmed PA [*n* = 107] or PA excluded [*n *= 293]). Thirty‐nine individuals with ‘possible PA’ diagnosis and 16 individuals with unknown final diagnosis were excluded from this analysis. Among the 400 individuals with confirmed outcome, primary and secondary care ARR data were available from 364 (secondary care ARR test results were not available in 36 individuals due to the test not being done or data missing). Among the 364 individuals included in this analysis, sensitivity in detecting confirmed PA was comparable between primary care (91.7% [95% CI 87.3–96.2]) and secondary care (92.1% [95% CI 87.2–97.1])) (*p* = 0.467). Similarly, negative predictive values were also comparable between primary care (93.6% [95% CI 90.0–97.1]) and secondary care (96.0% [95% CI 93.5–98.6]) (*p* = 0.191) (Supporting Table S2). Changing the laboratory assay methods in 2015 did not affect the comparability of primary and secondary care test results. Using different definitions for positive ARR result (i.e., including aldosterone or renin thresholds) did not change the test performance (Supporting Table S3). The percentage of individuals with false positive and false negative primary care ARR screening has varied over time with no clear trend (Supporting material Figure S3).

### 3.3 Factors Predicting PA During Primary Care Assessment

Predictors of final diagnosis were determined using data from 400 individuals with confirmed outcome (PA confirmed or PA excluded). In multivariable logistic regression analysis, hypertension, male sex, hypokalaemia and high ARR were independent predictors of confirmed PA (as opposed to no PA) (Table 3).

**Table 3:** Predictors of confirmed PA, as determined at the time of primary care assessment.

Predictors	Odds ratio (95% confidence interval)	*P* value
Age	1.0 (0.9–1.0)	0.124
Male sex	3.1 (1.7–5.6)	0.001
Hypertension	24.5 (3.2–188.0)	0.002
Hypokalaemia	3.6 (1.7–7.6)	< 0.001
Aldosterone renin ratio^a^	3.0 (2.2–4.2)	< 0.001

*Note:* Estimates derived from multivariable logistic regression modelling, including 400 individuals who had primary care assessment and final diagnosis of either confirmed PA or PA excluded.

Amongst individuals who were on antihypertensives, number of antihypertensives used were not different between the two groups: PA confirmed and PA excluded (1.5 ± 0.7 vs 1.5 vs ± 0.7, *p* = 0.886). On univariable logistic regression analyses among individuals who were taking antihypertensives, there was no association between PA status and use of ≥ 2 antihypertensives (*p* = 0.974) or use of ≥ 3 antihypertensives (*p* = 0.926).

a The relationship between diagnosis of PA and aldosterone renin ratio were nonlinear and therefore, natural log transformation of aldosterone renin ratio was used in the regression model. Odds ratio is for one unit of log2 (ARR).

Amongst biochemical tests performed in primary care, area under the ROC curve for diagnosing PA was statistically significantly higher for ARR (0.81, 95% CI 0.77–0.86) (Supporting Table S4 (a) and (b)). Aldosterone to renin ratio > 34.7 (sensitivity 87.9%, specificity 64.8%), aldosterone level > 418 pmol/L (Sensitivity 75.7%, specificity 51.9%), direct renin concentration < 21.9 mIU/L (sensitivity 88.8%, specificity 51.5%) and potassium level < 3.5 mmol/L (sensitivity 78.6%, specificity 48.0%) were the optimum independent cut‐off thresholds for predicting PA at primary care assessment (Supporting Table S5). (Note that results of direct renin concentration derived from iSYS assay in our reference laboratory are approximately equivalent to twice the value derived from most other assays [see Section 1 in Supporting material]).

On multivariable logistic regression analysis, after adjusting for age, sex, and use of different antihypertensive medications, use of beta‐blocker was associated with higher risk of false positive test result (OR 3.5, 95% CI 1.1–12.0, *p* = 0.042, when compared to true positive result). The association between false negative test result in primary care and use of ACEI/ARBs did not reach statistical significance (OR 3.6, 95% CI 0.8–16.7, *p* = 0.104 when compared to true negative result) (Supporting Tables S6 and S7). Diuretics did not show an association with false positive or false negative results, but only 8.7% of patients were on diuretics at the time of primary care testing. Three patients had secondary care tests done while on interfering medications (beta blockers) as it was not possible to stop the medication due to other compelling indications. Excluding them from the analyses did not alter the results.

## 4 Discussion

To our knowledge, this is the first UK study of effectiveness of biochemical screening for PA initiated in primary care. We show that it is possible to screen for PA in primary care and that this is occurring relatively frequently in Oxfordshire with 2915 individuals screened over a 14‐year period. Prevalence of PA in this population was 5.0% (3.7% with confirmed PA and 1.3% with possible PA). We found that 28% of patients identified with positive ARR in primary care were *not* referred to secondary care and hence missed the opportunity for further confirmatory testing and potential targeted treatment for PA. ARR performed in primary care showed comparable sensitivity and negative predictive value to secondary care testing with modest concordance. We found ARR of ≥ 30 pmol/mU to be a simple threshold with good sensitivity and negative predictive value for referring to secondary care for further assessment. Hence, we would argue that screening for PA with ARR should occur more widely in primary care with a clear threshold for referral given the recognition that a significant percentage of individuals with PA remain undiagnosed [7, 8, 9].

Extending screening for PA routinely to primary care would expand the population screened. Geographical inequities in screening, with higher screening rates in individuals residing closer to secondary care services are reported [20]. Routine primary care screening would overcome these inequities but also reduce the number of referrals to secondary care for screening of PA, with attendant cost savings. For example, one in two individuals referred to secondary care with suspected PA and positive ARR in primary care were diagnosed with confirmed (36.9%) or possible (14.1%) PA. If ARR was not available in primary care and assuming all individuals who were suspected to have PA were referred to secondary care, only one in 20 individuals would be diagnosed with confirmed (3.7%) or possible PA (1.3%), reflecting up to 18 avoidable referrals to secondary care for every person diagnosed with PA. In fact, primary care screening for PA is established in few other countries [13]. Having clear screening criteria, simple criterion for referral to secondary care (e.g.: an ARR ≥ 30 pmol/mU in our cohort) and raising awareness of the diagnosis of PA among primary care colleagues are all potential strategies to improve referral rates and case detection.

The 2016 Endocrine Society guidelines for case detection of PA recommend screening individuals with resistant hypertension, hypertension controlled with four or more antihypertensives, hypertension with hypokalaemia, hypertension with adrenal incidentaloma, hypertension with obstructive sleep apnoea or family history of young onset hypertension/stroke/primary aldosteronism [2]. In contrast, the 2024 Guidelines for hypertension by the European Society of Cardiology recommend that screening for PA should be considered in all adults with confirmed hypertension [21]. Our study found that male sex, presence of hypertension, hypokalaemia and raised ARR independently predicted increased risk of having PA. Additionally, we found that the prevalence of PA amongst those taking one, two or three antihypertensives were all ~30%, suggesting that a significant percentage of patients with primary aldosteronism, may not be identified by the Endocrine Society screening criteria which highlights the need for a more widespread screening strategy.

Although it is estimated that 10–15% of individuals with hypertension have underlying PA, the percentage diagnosed with PA in the population screened here is much lower (5%). Similarly low detection rates have been reported previously [13]. The large percentage of negative results suggests scope to refine patient‐selection criteria for screening. For instance, detection rate was low among individuals without hypertension (i.e. screened for isolated hypokalaemia or adrenal incidentaloma). In contrast, presence of hypertension with an additional predictive factor increased the detection rate. For example, in our cohort, the percentage diagnosed with PA was highest amongst those who were screened for hypertension and hypokalaemia (37%), or young‐onset hypertension (16%).

A recognised barrier to primary care PA screening has been the potential need to adjust antihypertensive medications [22]. In our study cohort, use of beta blockers was associated with higher risk of having a false positive test result. This emphasises the importance of endocrinologists working with primary care colleagues to help interpret the results in the context of medications used at the time of testing. For example, a positive test result while taking ACEI/ARB (which may give a false negative test result) would strongly raise the suspicion of PA, and should therefore be referred to secondary care for further assessment [23]. A converse scenario would be non‐suppressed renin while on a beta‐blocker making PA very unlikely. A positive ARR result with beta‐blocker use and a negative test result with ACEI/ARB use need to be interpreted with caution, as these could be false positive and false negative results respectively. Consistent communication with primary care around interpretation of ARR whilst on interfering medications is important and will help direct referral for further assessment when needed.

Limitations of our study include its retrospective design which may have introduced misclassification bias. We did not have data on individuals who had negative primary care ARR and were not referred to secondary care. Including their data would have allowed better definition of screening criteria and assessment of the need to repeat ARR in secondary care. Nevertheless, this ‘real‐world’ data provides valuable insights into the feasibility of PA screening in primary care. Furthermore, the number of individuals with PA is likely to be low in a cohort with negative ARR and therefore their inclusion would have been unlikely to affect final results. We did not compare primary care‐initiated screening strategy with secondary care‐initiated screening strategy, as all individuals included in our analysis entered screening via primary care referral. However, this cohort gave the unique opportunity to compare the performance of ARR as a screening tool in the two different settings. Comparing primary and secondary care ARR tests could be limited by biological variability and changes in medication between the tests. However, testing typically takes place in the morning (as samples need to be transported to the laboratory within 4 h of collection) in both primary and secondary care thus limiting the effect of timing on the test results. Secondly, we have analysed the effect of medication changes through regression models to minimise this bias. Finally, wider implementation of PA screening in primary care requires careful assessment of capacity of the primary care system to take on this workload. This was not addressed in this study as it was already routine practice in Oxfordshire. However we have since engaged with primary care colleagues to assess this potential limitation of the PA pathway.

In conclusion, our study demonstrates that primary care screening for PA is feasible, accurate and has modest concordance with secondary care testing. Sensitivity and negative predictive value of primary care ARR are high and comparable to that of secondary care. Detection rates could be improved by careful adherence to published screening criteria and the use of a simple referral criterion to secondary care such as a clear ARR threshold (ARR > 30 pmol/mU from our data set and assay parameters). Guidance around interpretation of the test when on potentially interfering antihypertensives as well as raised awareness of this diagnosis would improve referral rates and ultimately, effective targeted treatment for this underdiagnosed endocrine cause of hypertension.

## Conflicts of Interest

The authors declare no conflicts of interest.

## Supporting information

Supplementary_materials_R3_clean.

## Data Availability

The data that support the findings of this study are available from the corresponding author upon reasonable request.
