# Supplementary material for: Is It Possible to Screen for Primary Aldosteronism Effectively in Primary Care?
Source: Clin Endocrinol (Oxf). 2025 Apr 7;103(2):129–36. doi: 10.1111/cen.15247 (PMC12223710; doi:10.1111/cen.15247)
Supplement: Supplementary file 1 — Supplementary_materials_R3_clean. [file CEN-103-129-s001.docx]

**Supplementary material**

**Section 1**

**Clarification on direct renin concentration**

A number of direct renin concentration (DRC) methods are commercially available but due to a lack of consistent calibration approaches the results obtained differ^1^.

The DRC method in our hospital was the IDS i-Sys method (Immunodiagnostic Systems Limited, Boldon, UK) ^2^.

Internal method verification data included a comparison on patient samples (n=67) that showed that the relationship between the renin activity (range <0.2 to 24.0 pmol/L/hr) and the renin concentration (range 1.99 to 857.9 mIU/L) by immunoassay is demonstrated by the following formula (unpublished data):

iSYS Direct renin concentration [in mIU/L] = 23.80 x (plasma renin activity in pmol/L/hr) – 1.65.

This contrasts with other methods where direct renin concentration is approximately 8-12 times that of plasma renin activity^1^.

Therefore, DRC of 21.9 mIU/L in our assay is approximately equal to DRC ~ 10 mIU/L in other assays. Using this threshold provides consistency across time in our analysis.

**References**

1. Funder JW, Carey RM, Mantero F, et al. The Management of Primary Aldosteronism: Case Detection, Diagnosis, and Treatment: An Endocrine Society Clinical Practice Guideline. J Clin Endocrinol Metab. 2016;101(5):1889-1916. doi:10.1210/jc.2015-4061 *(please see table 6)*

2. O’Shea PM, Griffin TP, Browne GA, et al. Screening for primary aldosteronism using the newly developed IDS-iSYS® automated assay system. Pract Lab Med. 2017;7:6-14. doi:10.1016/j.plabm.2016.11.002

**Section 2**

**Tables**

**Table S1.** Medication use at the time of primary care ARR test.

| **Medication use at primary care testing** | **Patient group** | | |
| --- | --- | --- | --- |
|  | **Primary care ARR negative**  **(n=163)** | **Primary care ARR positive**  **(n=160)** | **P value** |
| Mineralocorticoid antagonist | 5 (3.0) | 6 (3.8) | 0.768 |
| Angiotensin converting enzyme inhibitors / angiotensin receptor blockers | 42 (25.6) | 43 (27.0) | 0.802 |
| Beta blockers | 12 (7.3) | 20 (12.6) | 0.138 |
| Alpha blockers | 29 (17.7) | 39 (24.5) | 0.172 |
| Dihydropyridine calcium channel blockers (eg: amlodipine) | 35 (21.3) | 60 (37.7) | 0.002 |
| Non-dihydropyridine calcium channel blockers (eg: diltiazem) | 1 (0.6) | 1 (0.6) | 1.000 |
| Thiazides | 17 (10.4) | 11 (6.9) | 0.324 |

Data presented are number (%). All individuals with data on medication use (irrespective of the number of medication) are included in the analysis. Comparisons were made using Chi square test.

**Table S2.** Performance of aldosterone-to-renin ratio (ARR) as a screening for detecting confirmed primary aldosteronism (PA) (n=364)^a^

|  | **Before and during 2015 (n = 77)** | | | **After 2015 (n = 287)** | | | **Combined (n = 364)** | | |
| --- | --- | --- | --- | --- | --- | --- | --- | --- | --- |
|  | **Primary care ARR** | **Secondary care ARR** | **P value** | **Primary care ARR** | **Secondary care ARR** | **P value** | **Primary care ARR** | **Secondary care ARR** | **P value** |
| Sen (%) | 81  (66-98) | 81  (62-100) | 0.655 | 93  (88-98) | 95  (90-100) | 0.527 | 92  (87-96) | 92  (87-97) | 0.467 |
| Spec (%) | 59  (47-71) | 80  (70-90) | 0.005 | 60  (54-66) | 80  (74-85) | <0.001 | 60  (54-65) | 80  (75-85) | <0.001 |
| PPV (%) | 41  (26-55) | 52  (32-71) | 0.013 | 46  (39-54) | 62  (53-71) | <0.001 | 53  (47-59) | 65  (58-73) | <0.001 |
| NPV (%) | 90  (82-99) | 94  (88-100) | 0.421 | 96  (92-99) | 98  (95-100) | 0.323 | 94  (90-97) | 96  (94-99) | 0.191 |
| PDLR | 2.0  (1.4-2.9) | 4.1  (2.4-7.2) | 0.013 | 2.3  (1.9-2.7) | 4.7  (3.6-6.1) | <0.001 | 2.3  (2.0-2.6) | 4.6  (3.6-5.8) | <0.001 |
| NDLR | 0.3  (0.1-0.8) | 0.2  (0.8-0.7) | 0.406 | 0.1  (0.1-0.3) | 0.1  (0.1-0.2) | 0.313 | 0.1  (0.1-0.2) | 0.1  (0.1-0.2) | 0.179 |

Data presented are estimates and 95% confidence intervals within brackets. ^a^ analysis based on individuals who had ARR performed in both primary and secondary care and diagnosis confirmed as PA or definitively excluded as not having PA (ie excluding possible PA).

*ARR: secondary care aldosterone renin ratio test, NDLR: negative diagnostic likelihood ratio, NPV: negative predictive valuePDLR: positive diagnostic likelihood ratio, PPV: positive predictive value, Sen: Sensitivity, Spec: Specificity*

**Table S3.** Performance of aldosterone renin ratio test performed in primary and secondary care in detecting confirmed primary aldosteronism, with varying criteria to define a positive ARR result (n = 364)

Analysis is based on 364 individuals who had ARR done in both primary and secondary care and final diagnosis was confirmed PA or PA excluded. Data in brackets are 95% confidence intervals.

*ARR: aldosterone to renin ratio, NLR: negative likelihood ratio, NPV: negative predictive value, PLR: positive likelihood ratio, PPV: positive predictive value*

**Table S4(a)**. Area under ROC curve for different biochemical tests performed at primary care in predicting confirmed primary aldosteronism

| **Test** | **Area under ROC curve (95% confidence interval)** | | |
| --- | --- | --- | --- |
|  | **Before and during 2015**  **(n = 86)** | **After 2015**  **(n = 314)** | **Total population (n = 400)** |
| Aldosterone:renin ratio | 0.82 (0.73-0.91) | 0.84 (0.808-0.999) | 0.81 (0.77-0.86) |
| Aldosterone | 0.70 (0.66-0.82) | 0.77 (0.606-0.73) | 0.77 (0.61-0.73) |
| Direct renin concentration | 0.77 (0.54-0.88) | 0.77 (0.71-0.81) | 0.74 (0.77-0.88) |
| Potassium | 0.60 (0.41-0.92) | 0.63 (0.60-0.68) | 0.65 (0.57-0.74) |

Data presented are the estimates and 95% confidence intervals. Analysis is based on 400 individuals who had primary care screening ARR and had either confirmed primary aldosteronism or no primary aldosteronism (i.e., primary aldosteronism excluded)

**Table S4(b).** P values for comparison of area under the ROC curve for different biochemical tests performed at primary care in predicting confirmed primary aldosteronism

|  | **Aldosterone:renin ratio** |  |  |
| --- | --- | --- | --- |
| **Aldosterone** | < 0.001 | **Aldosterone** |  |
| **Direct renin concentration** | 0.005 | 0.038 | **Direct renin concentration** |
| **Potassium** | <.001 | 0.792 | 0.008 |

**Table S5** Primary care test performance characteristics based on current cut offs and optimum cut-offs (determined with Youden Index) ^a^

|  | **Current practice** | | | **Optimal** | | |
| --- | --- | --- | --- | --- | --- | --- |
|  | **Cut off** | **Sen (%)** | **Spec (%)** | **Cut off** | **Sen (%)** | **Spec (%)** |
| Aldosterone to renin ratio (pmol/mU) | ≥30 | 91.7 | 58.4 | 34.7 | 87.9 | 64.8 |
| Aldosterone (pmol/L) | ≥400 | 76.6 | 49.1 | >419 | 75.7 | 51.9 |
| Renin (mIU/L) | <20 | 84.1 | 53.2 | <22 | 88.8 | 51.5 |
| Potassium (mmol/L) | - | - | - | <3.5 | 78.6 | 48.0 |

^a^ 400 individuals with primary care test result and final diagnosis of confirmed or excluded PA included in the analysis

Aldosterone in pmol/L, renin in mU/L, potassium in mmol/L, aldosterone to renin ration in pmol/mU

*AUC: areas under ROC curve, Sen: sensitivity, Spec: specificity*

**Table S6** Determinants of the 81 false positives among the 144 people with a positive aldosterone:renin ratio test in primary care *

|  | **Odds ratio** | **p value** |
| --- | --- | --- |
| Age (years) | 1.02 (0.99-1.05) | 0.361 |
| Male sex | 0.26 (0.12-0.58) | 0.001 |
| Mineralocorticoid receptor antagonist | 0.00 (0.00-0.00) | 0.999 |
| ACE inhibitors / Angiotensin receptor blocker | 0.20 (0.08-0.52) | 0.001 |
| Beta blocker | 3.55 (1.05-12.03) | 0.042 |
| Alpha blocker | 0.44 (0.18-1.10) | 0.080 |
| Dihydropyridine calcium channel blocker | 0.51 (0.24-1.09) | 0.080 |
| Thiazide diuretic | 0.96 (0.24-3.80) | 0.260 |

* A false positive primary care ARR test result defined as primary care ARR result of ≥ 30 pmol/mU and PA was excluded. Individuals with possible PA diagnosis were excluded from the analysis. Data from 144 individuals with established diagnosis, medication data and positive primary care ARR test result included in the analysis. Among them, 81 had false positive primary care aldosterone to renin ratio test.

*ACEI: angiotensin converting enzyme inhibitor, ARB: angiotensin receptor blocker*

**Table S7** Determinants of the 8 false negatives among the 159 people with a negative screening in primary care with the aldosterone renin ratio *

|  | **Odds ratio** | **p value** |
| --- | --- | --- |
| Age (years) | 1.02 (0.96-1.09) | 0.440 |
| Male sex | 2.50 (0.44-14.08) | 0.298 |
| Mineralocorticoid receptor antagonist | 8.26 (0.52-130.6) | 0.134 |
| ACE inhibitors / Angiotensin receptor blocker | 3.58 (0.77-16.61) | 0.104 |
| Beta blocker | 0.00 | 0.999 |
| Alpha blocker | 2.06 (0.33-12.95) | 0.441 |
| Dihydropyridine calcium channel blocker | 1.18 (0.21-6.73) | 0.851 |
| Thiazide diuretic | 0.82 (0.08, 8.40) | 0.864 |

* A false negative is defined as a negative primary care ARR test (ARR < 30 pmol/mU) and final diagnosis being confirmed PA. Individuals with possible PA diagnosis were excluded from the analysis. Data from 159 individuals with established diagnosis, medication data and negative GP ARR test result included in analysis. Among them, eight had false negative primary care aldosterone to renin ration test result.

*ARR: aldosterone renin ratio, PA: primary aldosteronism*

**Section 3**

**Figures**

**
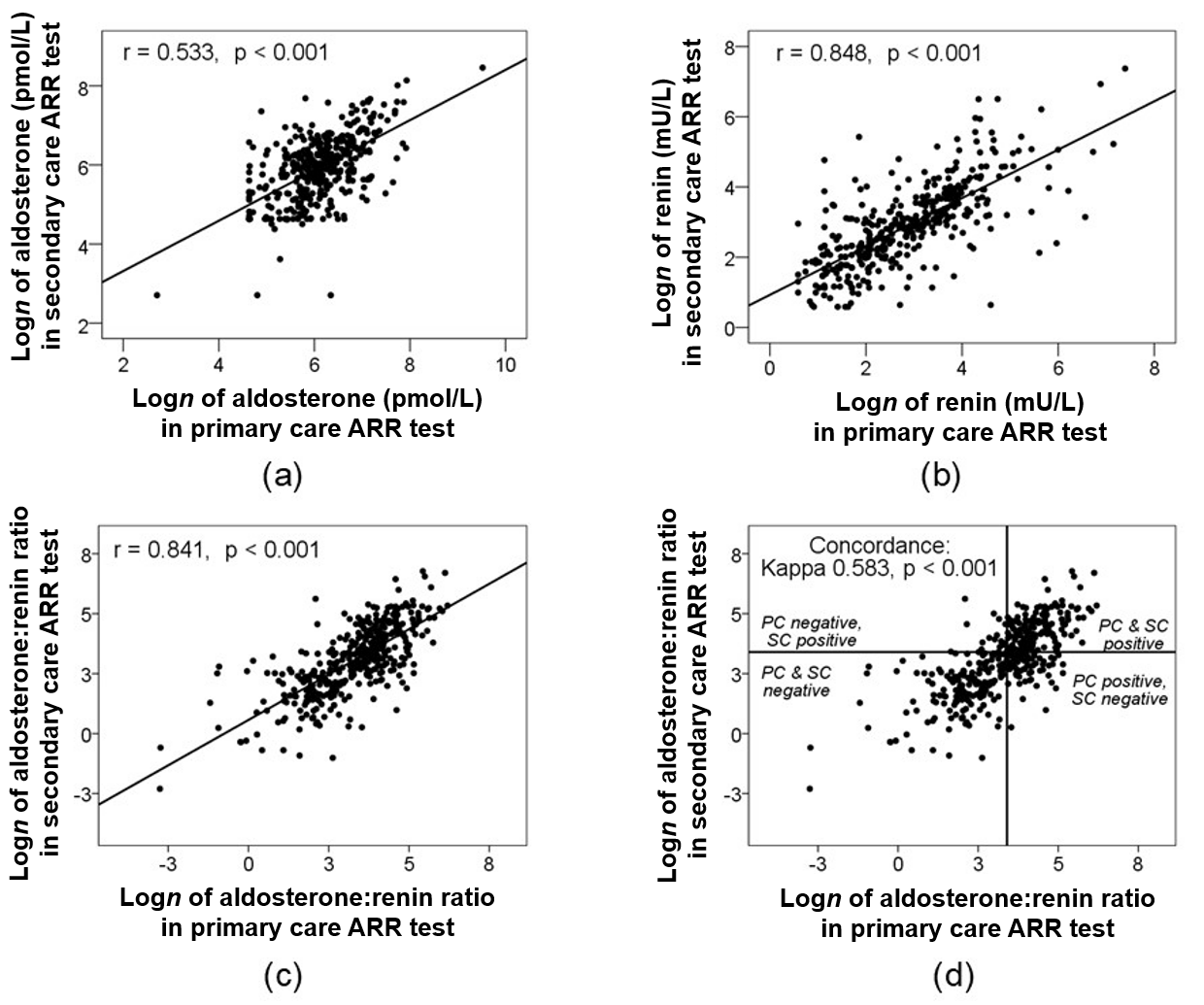
**

**Figure S1.** Associations and Spearman correlation coefficients of (a) aldosterone, (b) renin and (c) aldosterone renin ratio performed in primary and secondary care settings and (d) concordance between aldosterone renin ratio test results performed in primary and secondary care settings. A ‘positive’ test result defined as aldosterone renin ratio ≥ 30 pmol/mU (reference lines). Analysis is based on data from 394 individuals who had ARR performed in both primary and secondary care settings (irrespective of the final outcome of diagnostic evaluation).

Values are expressed after Logn transformation (logarithm defined to base 2) for clarity of graphs.

*ARR: aldosterone:renin ratio, PC: primary care, SC: secondary care*


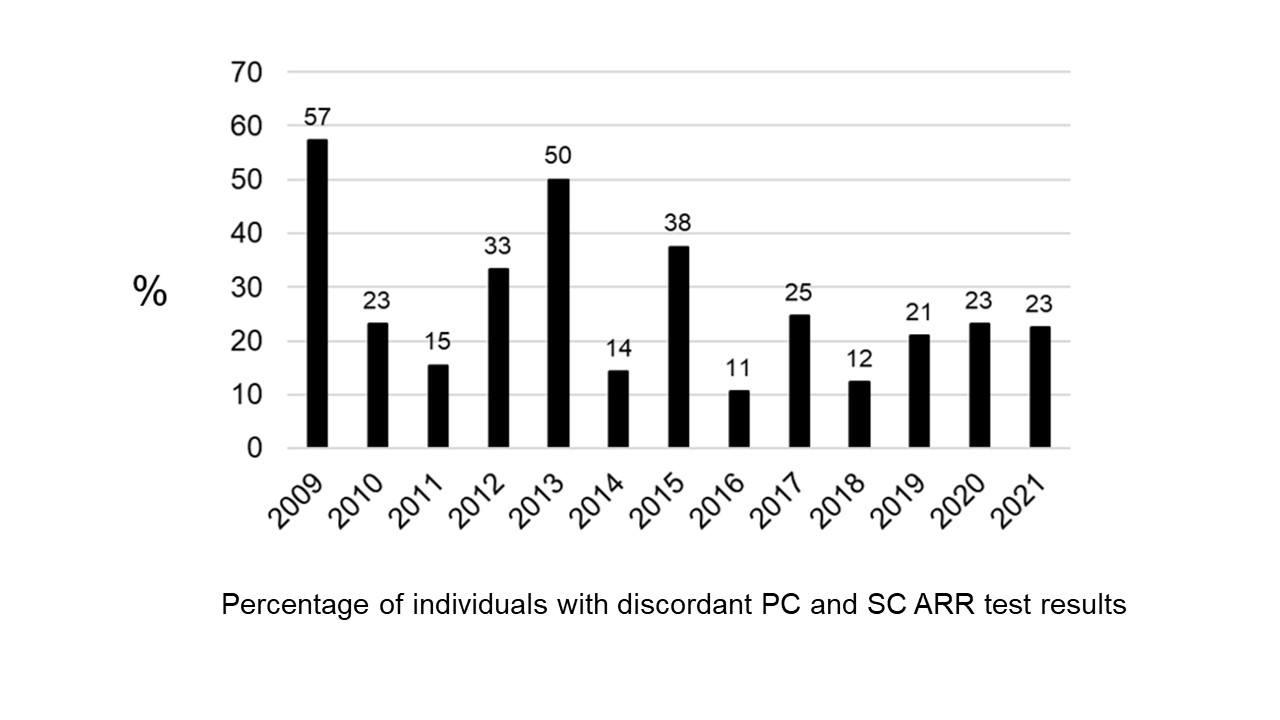


Year

**Figure S2.** Percentage of individuals with discordant ARR test results in primary and secondary care testing each year from 2009 to 2021 (among 394 individuals who had ARR performed in both settings). Positive test defined as ARR ≥ 30 pmol/mU.

*ARR: aldosterone renin ratio*


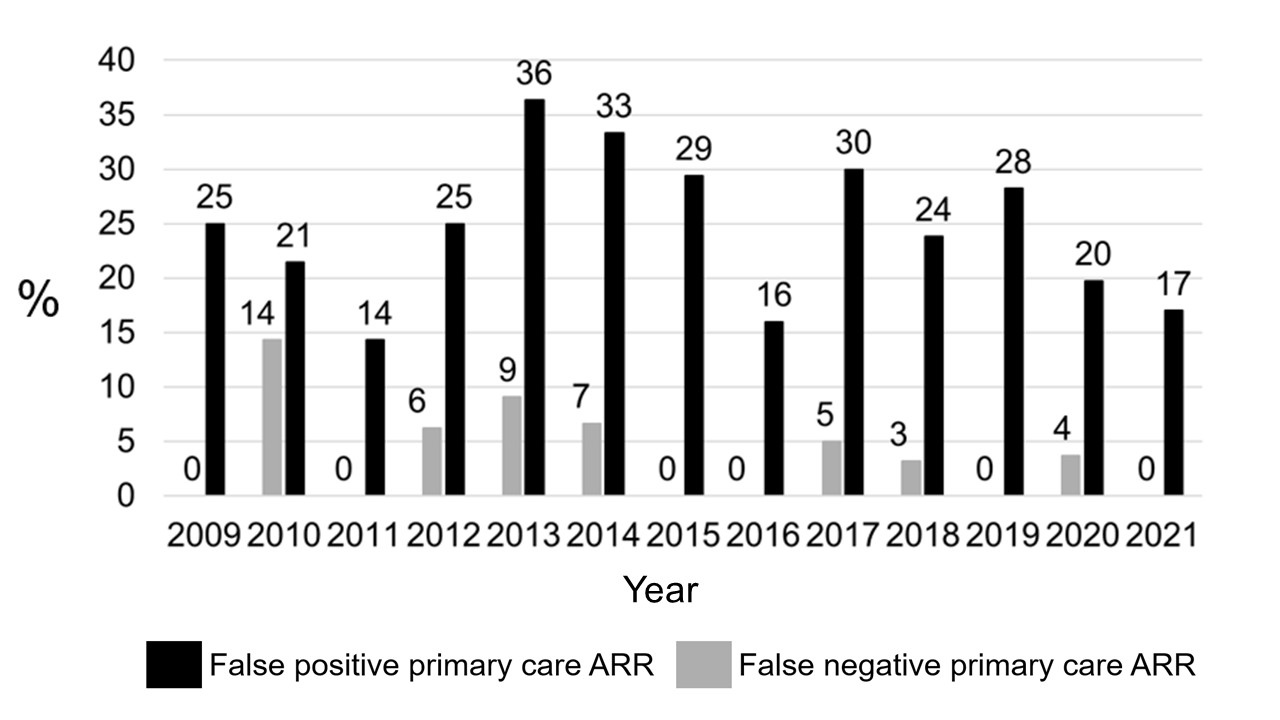


**Figure S3.** Percentage of individuals with false positive or false negative primary care aldosterone renin ratio test results each year from 2009 to 2021 (among 439 individuals with known outcome). Positive test defined as ARR ≥ 30 pmol/mU.

*ARR: aldosterone renin ratio*
